# Draft Genome Sequence and Annotation of the Entomopathogenic Bacterium *Xenorhabdus szentirmaii* Strain DSM16338

**DOI:** 10.1128/genomeA.00190-14

**Published:** 2014-03-13

**Authors:** Maxime Gualtieri, Jean-Claude Ogier, Sylvie Pagès, Alain Givaudan, Sophie Gaudriault

**Affiliations:** a Nosopharm, Nîmes, France; b INRA, UMR Diversité, Génomes et Interactions Microorganismes-Insectes (DGIMI), Montpellier, France; c Université Montpellier 2, UMR Diversité, Génomes et Interactions Microorganismes-Insectes (DGIMI), Montpellier, France

## Abstract

We report the genome sequence of *Xenorhabdus szentirmaii* DSM16338 (4.84 Mb), a symbiont of the entomopathogenic nematode *Steinernema rarum*. This strain produces antimicrobial activity.

## GENOME ANNOUNCEMENT

*Xenorhabdus* is a symbiont of nematodes of the family *Steinernematidae*, pathogenic for a wide variety of insects (1). The entomopathogenic nematodes are used as biological control agents for soil-inhabiting insects (2). The *Xenorhabdus* genus is also a source of secondary metabolites (3). These metabolites are bioactive molecules with a broad spectrum of potential functions, such as insecticidal, antitumor, and antimicrobial activities. In the course of antimicrobial screening on culture supernatants of a collection of *Xenorhabdus* strains, we identified *Xenorhabdus szentirmaii* DSM16338 as an important producer of antimicrobial activity, a property previously described by other authors (4–6).

We sequenced *Xenorhabdus szentirmaii* DSM16338, a symbiont of the entomopathogenic nematode *Steinernema rarum* from Argentina (7). The genomic DNA was purified from our laboratory stock according to the method of Brenner et al. (8). The sequencing strategy was conducted by GATC Biotech (Konstanz, Germany). We used a mixed sequencing strategy with Roche 454 GS-FLX titanium and Illumina technologies. Sequencing of a 450-nucleotide mate-paired library with a GS FLX sequencer resulted in 271,899 reads with a median length of 334 nucleotides. Semiautomatic GS FLX assembly generated 169 contigs comprising a total length of 4.82 Mb. Sequencing of a 3-kb paired-end library with an Illumina HiSeq 2000 sequencer (read length: 2 × 50 nucleotides) resulted in 40,772,101 read pairs that were used for mapping against the GS FLX data with homopolymer correction. The final assembly consisted of 164 contigs comprising a total length of 4.84 Mb (4.82 Mb without undetermined bases) and has a 43.98% GC.

Functional annotation was carried out using tools of the MicroScope platform (9). The annotated genomes were implemented in the public XenorhabduScope database (https://www.genoscope.cns.fr/agc/microscope/home/index.php). The assembly of *X. szentirmaii* DSM16338 contains 4,794 genomic objects, including 4,680 coding sequences, 4 rRNA genes, 58 tRNA genes, and 23 noncoding RNAs. Genome annotation highlighted the presence of 71 genes encoding nonribosomal peptide synthetases and polyketide synthases in *X. szentirmaii* DSM16338. Therefore, this bacterium is a promising reservoir for nonribosomally synthesized peptides with new bioactive effects, such as antimicrobial activities. Further genomic analyses will be performed to identify gene clusters for biosynthesis of antimicrobial molecules.

### Nucleotide sequence accession numbers.

This whole-genome shotgun project has been deposited at EMBL under the accession no. CBXF000000000. The version described in this paper is the first version, CBXF010000000.
